# 

**Published:** 1896-04-18

**Authors:** 


**Notices of Births, Deaths, and Marriages** are inserted in this column at a charge of 2s. 6d. for an announcement not exceeding four lines.

### NOTICE TO CORRESPONDENTS.

All MS., Letters, Books for Review, and other matters intended for the Editor should be addressed The Editor, The Lodge, Porchester Square, London, W.

The Editor cannot undertake to return rejected MS., even when accompanied by stamped directed envelope.

**Notice to Advertisers and Subscribers.**

All Advertisements, Orders for Copies of The Hospital and Business Communications should be addressed to **THE MANAGER, (Not to The Editor),** The Hospital, 428, Strand, London, W.C.

The Cost of Subscription, post free, is as follows:—Per week, 2½d.; per quarter, 2s. 8d.; per half-year, 5s. 3d.; per annum, 10s. 6d. Foreign:—Per Annum, 15s. 2d.; payable, in all cases, in advance.

Ready Shortly.

**"Burdett's Hospitals and Charities, 1896."**

Seventh year of publication. Over 1,000 pp. Scarlet cloth, gilt lettered. Price 5s. A most complete guide to British, Colonial, Indian, and American Hospitals, Dispensaries, Nursing and Convalescent Institutions, and Asylums. A few complete sets of the preceding six issues of the "Annual" may still be obtained on application to the Publishers, The Scientific Press (Limited), 428, Strand, London, W.C.

**NOTICE.—Vol. XIX. of "The Hospital" (October, 1895, to March, 1896), in handsome cloth binding, is now ready and on sale at the Office of this Journal. Price 7s. 6d. Cases for binding the Numbers contained in this Volume, 1s. 6d. Index to Vol. XIX., 2½d., post free.**

**The Monthly Part for April is now ready, price 6d., by post, 8d.**

### THE STUDENT OF MEDICINE.

### APPOINTMENTS.

S. J. Aarons, M.B., C.M., appointed Non-resident Clinical Clerk to the Edinburgh Royal Infirmary. H. P. Bennet, M.B., C.M., appointed Non-resident Clinical Clerk to the Edinburgh Royal Infirmary. C. J. Bennett, M.R.C.S.Eng., appointed Medical Officer of Health to the Disley and Hayfield Rural District Councils. J. G. Cattanach, M.B., C.M., appointed Non-resident Clinical Clerk to the Edinburgh Royal Infirmary. M. Christie, M.B.Lond., appointed Junior Medical Officer of the Greenwich Workhouse. T. Coghlan, L.R.C.S.I., L.M., appointed Medical Officer for the Kilmacow Dispensary. J. T. D. Crinion, L.R.C.P., L.R.C.S.I., appointed Medical Officer for the Rathmore Dispensary District. R. W. Cunningham, M.B., C.M., appointed Resident Surgeon to the Edinburgh Royal Infirmary. G. Dickson, M.D., appointed Non-resident Clinical Clerk to the Edinburgh Royal Infirmary. J. S. Fowler, M.B., C.M., appointed Non-resident Clinical Clerk to the Edinburgh Royal Infirmary. F. G. Gardner, M.R.C.S., &c., appointed Medical Officer to the Countess of Warwick's Home for Crippled Children. C. Giggs, F.R.C.S., appointed Assistant Surgeon to Charing Cross

THE STUDENT OF MEDICINE—*continued.*

Hospital. A. B. Giles, M.D., appointed Non-resident Clinical Clerk to the Edinburgh Royal Infirmary. J. Gray, M.A., M.B., C.M., appointed Resident Surgeon to the Edinburgh Royal Infirmary, J. H. Henderson, M.B., C.M., appointed Non-resident Clinical Clerk to the Edinburgh Royal Infirmary. R. J. Johnston, M.B., C.M., appointed Non-resident Clinical Clerk to the Edinburgh Royal Infirmary. H. G. P. Le Fanu, L.R.C.S., L.R.C.P.I., appointed Surgeon to the Derbyshire Hospital for Children. G. R. Livingston, M.B , C.M., appointed Resident Physician to the Edinburgh Royal Infirmary. D. Macaulay, M.B., C.M., appointed Non-resident Clinical Clerk to the Edinburgh Royal Infirmary. K. McLean, M.B., C.M., appointed Resident Surgeon to the Edinburgh Royal Infirmary. N. H. MacMillan, M.B., C.M., appointed Resident Physician to the Edinburgh Royal Infirmary. J. S. Martin, M.B., C.M., appointed Non-resident Clinical Clerk to the Edinburgh Royal Infirmary. R. L. Moorhead, M.B., C.M., appointed Resident Surgeon to the Edinburgh Royal Infirmary. W. R. Nattlo, M.R.C.S.Eng., L.R.C.P.Lond., appointed Medical Officer for Mafeking, Basutoland, South Africa. J. Orr, M.B., C.M., appointed Non-resident Clinical Clerk to the Edinburgh Royal Infirmary. A. J. Park, M.B., C.M., appointed Non-resident Clinical Clerk to the Edinburgh Royal Infirmary. H. Richardson, M.B., C.M., appointed Resident Surgeon to the Edinburgh Royal Infirmary. J. I. Sankey, M.R C.S., L.R.C.P., L.S.A., appointed Medical Officer and Public Vaccinator for the No. 6 (Brenchley) District of the Tonbridge Union. P. R. W. de Santi, F.R.C.S., appointed Assistant Surgeon and Aural Surgeon to the Westminster Hospital. J. Scott, M.B., C.M.Edin., appointed Medical Officer to Her Majesty's Prison, Holloway. R. P. Shearer, M.B., C.M.Glasg., appointed Medical Officer for the Gotham District of the Basford Union. H. J. F. Simson, M.B., C.M., appointed Resident Physician to the Edinburgh Royal Infirmary. W. M. Taylor, M.B., C.M., appointed Resident Physician to the Edinburgh Royal Infirmary. A. L. Turner, M.D., F.R.C.S.E., appointed Clinical Clerk to the Edinburgh Royal Infirmary. I. O. Veitch, M.B , C.M., appointed Non-resident Clinical Clerk to the Edinburgh Royal Infirmary. F. H. Waddy, M.B., C.M., appointed Non-resident Clinical Clerk to the Edinburgh Royal Infirmary. D. W. Watson, M.B., C.M., appointed Non-resident Clinical Clerk to the Edinburgh Royal Infirmary. J. Watt, M.B., C.M., appointed Resident Physician to the Edinburgh Royal Infirmary. S. R. Wells, M.D., B.Sc.Lond., M.R.C.P., appointed Medical Registrar to St. George's Hospital. A. R. Wilson, M.A., M.B., C.M., appointed Resident Physician to the Edinburgh Royal Infirmary.

### VACANCIES.

The following vacancies are announced this week, the amount of salary in each case being given after the name of the institution:—

### Resident Medical Officers.

Donegal District Lunatic Asylum, Letterkenny.—Assistant Medical Officer; qualified in medicine, surgery, and midwifery; unmarried, and not more than 30 years old. Salary £100 per annum, with furnished apartments, rations, washing fuel, light and attendance, valued at £100 per annum. Applications to Dr. Moore, Resident Medical Superintendent, by May 9th.

East London Hospital for Children and Dispensary for Women, Glamis Road, Shadwell.—Resident Medical Officer, doubly qualified. Salary, £80 per annum, with board and residence. Applications to Thomas Hayes, Secretary by April 18th.

General Hospital, Birmingham.—Assistant House Surgeon. Appointment for six months. No salary, but residence, board, and washing provided. Applications to Howard J. Collins, House Governor, by April 25th.

Halifax Infirmary and Dispensary.—Assistant House Surgeon; unmarried; doubly qualified and registered. Salary £50 per annum, with residence. board, and washing. Applications to Oates Webster, Secretary, by April 22nd.

Hereford County and City Asylum.—Medical Superintendent. Salary £400 per annum, with furnished house, coals, gas, vegetables, and washing. Applications to the Chairman, Asylum Committee, Shirehall, Hereford, by April 28th.

St. Mary's Hospital, Quay Street, Manchester.—Resident Medical Officer. Appointment for six months, subject to re election. Salary £70 per annum, with board and residence. Applications to the Chairman of the Board of Management by April 24th.

Sussex County Hospital, Brighton.—Fourth Resident Medical Officer; unmarried, and under 30 years of age. Emoluments are a salary not exceeding £30 per annum, with board, washing, and residence. Applications to the Secretary by April 22nd.

Wrexham Infirmary and Dispensary.—House Surgeon. Salary £80 per annum, with furnished rooms, gas, coal, and attendance. Applications, upon forms to be obtained from the Secretary, to be addressed to Mr. George Whitehouse, 27, Regent Street, Wrexham, by April 22nd.

### Non-Resident Medical Officers.

Bath Urban Sanitary Authority.—Medical Officer of Health. Appointment for one year. Salary, £200 per annum. Applications addressed to the Chairman of the Sanitary Committee to be sent to F. H. Moger, Clerk, 9, Wood Street, Bath, by April 22nd.

Dental Hospital of London and London School of Dental Surgery, Leicester Square.—Lecturer on Mechanical Dentistry. Applications to Morton Smale, Dean, by May 11th.

Hospital for Sick Children, Great Ormond Street, Bloomsbury.—Surgical Registrar. Appointment for one year. Honorarium £40. Applications to the Secretary by April 28th.

Owens College, Manchester.—Junior Demonstrator in Anatomy. Applications to the Registrar by April 27th.

Owens College, Manchester.—Junior Demonstrator of Physiology. Applications to the Registrar by April 25th.

Royal Orthopædic and Spinal Hospital, Birmingham.—Honorary Assistant Surgeon. Applications to E. J. Abbott, Honorary Secretary, 9, Bennett's Hill, Birmingham, by April 18th.

